# Early permanent dental eruption in obese/overweigh schoolchildren

**DOI:** 10.4317/jced.58568

**Published:** 2022-02-01

**Authors:** Carla Traver-Ferrando, Jorge Barcia-González

**Affiliations:** 1 Department of Pediatric Dentistry, Catholic University of Valencia ¨San Vicente Mártir ¨; 2 Doctoral School, Catholic University of Valencia ¨San Vicente Mártir ¨; 3 Department of Anatomy and Physiology. Catholic University of Valencia ¨San Vicente Mártir ¨

## Abstract

**Background:**

The WHO defines obesity as abnormal excessive fat accumulation. Indeed, it is the most relevant nutritional disorder in the developed countries. Nutrition turns out to be relevant for childhood development involving different systems and organs as and including teeth development. Prediction of teeth eruption results of relevance in odontopediatrics and more particularly at transitional stages with mixed dentition. Early teeth eruption can lead to dental defects and eventually to periodontal disease. In addition to this, obesity increases chronic periodontitis risk on teenagers and young-adults. Objectives: The aim of the work is to confirm the relation of weigh and dental eruption on six years old children.

**Material and Methods:**

519 six years old children were included on the analytic observational study. Clinical examination was conducted at school, weight and size was registered for BMI index (Kg/cm2). Permanent teeth presence was recorded during examination.

**Results:**

Overweight or obesity significantly doubles the probability to find both, the first permanent molars and the lower central incisors. In addition, we report herein a significant association between weight at birth and the first milk tooth eruption.

**Conclusions:**

Early dental eruption is observed in obese/overweight school children compared to normal weigh children.

** Key words:**Pediatric obesity, tooth eruption, permanent dentition.

## Introduction

The World Health Organization (WHO) defines obesity and overweight as abnormal and excessive fat accumulation harmful to health (1,2). Childhood and youth obesity is the most relevant nutritional disorder today, affecting the health of those suffering it in the present and in the future (3). While its prevalence has increased in the recent years, it is also concerning its progressive presence at younger ages (4,5). Obese children have a high probability of being obese adults with secondary alterations, such as cardiovascular disease, arterial hypertension, type 2 diabetes mellitus, dyslipidemia (1,5,6), and immune system alterations (1).

Given the multiplicity of factors involved in dental eruption, it is evident that the child`s overall development has a significant impact on such dental eruption (2). It is essential to know the age and sequence of eruption for each tooth, since eruptive alterations could condition the implementation of dental treatment strategies (7).

Alterations in tooth eruption can significantly impact oral health, due to their potential to cause occlusion defects (7-10) and crowding, leading to poor oral hygiene and periodontal disease (6,9,10). Obesity and overweight are considered a risk factor for periodontal disease in young adolescents and early adulthood (6,10,11).

Studies by Wise *et al*. show that childhood obesity and teething are positively correlated (12,13). In a cross-sectional study assessing the difference between chronologic age and dental age increased significantly with body mass index (BMI) (13). These results are in line with those of Eid *et al*., who also found a significant correlation between dental maturity and BMI (14).

The age at which each tooth erupts, eruptive sequence and its relationship to physiological parameters (resorption of the roots of deciduous teeth and exfoliation of deciduous teeth) have contributed to further research in this field. During the formation phases, the tooth is sensitive to systemic influences. Once formed and erupted, it is less sensitive to these changes (7). Therefore, we can hypothesize that 6-year-old children with high BMI present time differences on permanent tooth eruption.

Most of the data on BMI and dental eruption were performed on western countries Laos, Indonesia, Pakistan and even USA but little is known about this from Europe where culture, health systems, life styles are so diverse compared to these countries.

This work analyzed the presence of the first permanent molars and the lower central incisors in 6-year-old school children with high BMI compared with normal weight group. Other variables were also analyzed.

## Material and Methods

Cross-sectional and observational study was carried out in 519 6-year-old schoolchildren from 11 schools in Castellón de la Plana (Valencian Community) form October 2016 to April 2017).

Permissions were requested to the Education Research, Culture and Sports Council, of the Generalitat Valenciana. In addition, the study was approved by the Research and Ethics Committee at Catholic University of Valencia ¨San Vicente Mártir¨.

Sample size was estimated to detect a significant difference of 0,5 years (sig. 0.05, 0.8 power). Since the studied groups present different proportions sample sizes are different.

Moreover, as sample data collection was performed along school and classrooms the sample size was done by cluster sampling because schoolchildren on the same college can share common aspects. Interclass correlation coefficient quantified the relation level within groups finally giving n=325 normal weigh and n=73 overweigh as sample size.

The initial intention was to make an exhaustive sampling via e-mail inviting every school to take part in the study. Unfortunately, some schools declined the invitation and convenience sampling was finally performed. Parents were then invited to participate in the study by submitting informed consent and a questionnaire through the school. This questionnaire included information on sociodemographic variables, medical and dental history. This questionnaire collected information on the variables to be analyzed with the body mass index. Children with chronic syndromes or other pathologies potentially affecting dental eruption were excluded from the study.

Rejection participation rate by parents was 42. In addition, three students were excluded from the study because of lack of including criteria.

-Subjects

Clinical examination and dental check-up was individually performed. All the subjects were checked once by one examiner. Weight and height were obtained on barefoot children without heavy clothing for BMI. BMI classification was based on WHO percentile tables (4,15). Normal weight (15th and 85th percentile), overweight (85th and 95th percentile), and obesity (>97th percentile) were considered.

First upper and lower permanent molars and lower central incisors (4.6, 3.6, 2.6, 1.6, 3.1 y 4.1) were explored. Federation Dentaire Internationale (FDI) dental notation was used (16). Erupted tooth was considered when all or part of the crown was observed in the oral cavity.

The quantitative variables recorder were age, weeks of pregnancy 0:less than 37 weeks, 1: more than 37 weeks), weight at birth (0: less than 2.500 grams, 1: between 2.500-4.000 grams, 2: more 4.000 gram) and first temporary tooth eruption (0: <4 months, 1: from 4 - 9 month, 2: >9months), and also qualitative variables: Breastfeeding (0: <3 months, 1: From 3 to 6 months, 2: 6 - 12 months, 3: 12 - 18 months, 4: > 18 months), and the presence of obesity in any relative (0: none, 1: Father/mother (one of the two), 2: Father and mother (both), 3: Siblings).

-Statistic analysis

A descriptive analysis of the variables was carried out including the mean and standard deviation (SD) for continuous variables. Absolute and relative frequencies were described for the variables. t-test and one-way ANOVA test were used for the comparison between continuous variables. Chi-square test was used for the comparison between categorical variables.

The effect of BMI on dental eruption was analyzed taking into account potential confounding variables such as age of eruption of the first milk tooth, weight at birth, gestational age, lactation type and family obesity history. The Odds Ratio (OR) was expressed with 95% confidence interval. Significance was set at *p*<0.05. The SPSS statistical package (version 22) was used for statistical analysis.

Multiple binary logistic regression model for tooth eruption according to sex and age was estimated. This model estimates rate and 95% confidence Interval Odd ratio (OR). Additionally, binary logistic regression model with generalized estimating equations was included.

The homogeneity of the groups was checked according to the demographic profile (sex and age). Considering together the obese + overweight groups does not lead to heterogeneity. 47 overweight and 73 obese children were initially obtained, compared to 399 normal weight. The overweight + obese group increased the statistical power when compared to the normal weight group in all comparisons.

## Results

The sample consists of 267 men (51,4%) and 252 women (48,6%). 76,8% of the schoolchildren, without sex differences, were classified as normal weight, 9,1% overweight and 14,1% obese. Table 1 shows the distribution of the participants according to the school and weight group.

**Table 1:**
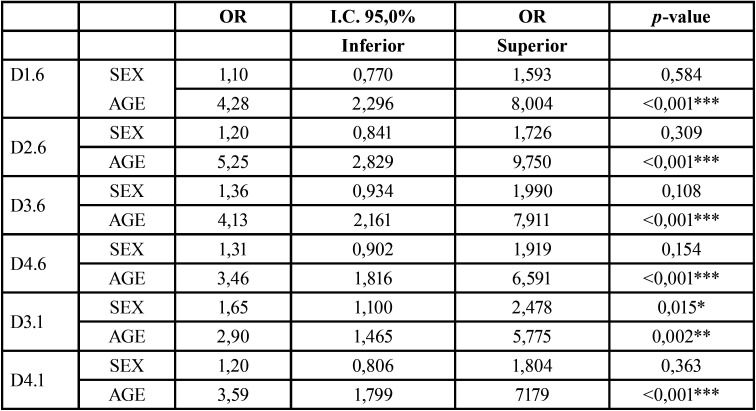
Probability of eruption by teeth.

Both overweight and obesity significantly increases the probability of early (precocious) emergence of the studied teeth. A child on this condition increases 1.5 to 2 times the probability of early permanent tooth eruption with respect normal weight children.

Multivariate analysis adjusted for sex and age indicated that obese or overweight groups were twice as likely to have the first upper permanent molars (1.6 and 2.6), the first upper left permanent molar (4.6) and the lower central incisors (3.1 and 4.1) than controls.

Overall, age is the most important factor. The estimated odds ratio ranges from 2.90 to 5.25. The probability of eruption is multiplied by 1.11 and 1.18 for each additional month. In all studied teeth, the impact of obesity on dental eruption remains sTable for the different age ranges within the analyzed time (Table 1).

As shown in Table 2, overweigh schoolchildren increased almost two-fold the probability to present erupted teeth (OR=1,88) compared to normal weigh ones (*p*=0,001). This is observed in any individual fitting age and sex. This significant effect is attribuTable to age (*p*<0,001). When analyzing the role of BMI on tooth eruption, the probability that all the permanent studied teeth were found increased with both BMI and age (Table 2).

**Table 2:**
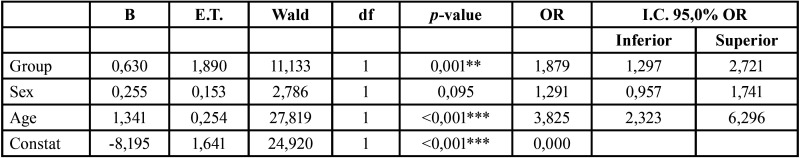
Probability of tooth eruption according to sex and age and group.

Weight at birth and first temporary tooth eruption are the most relevant variables explaining dental eruption (Table 3).

**Table 3:**
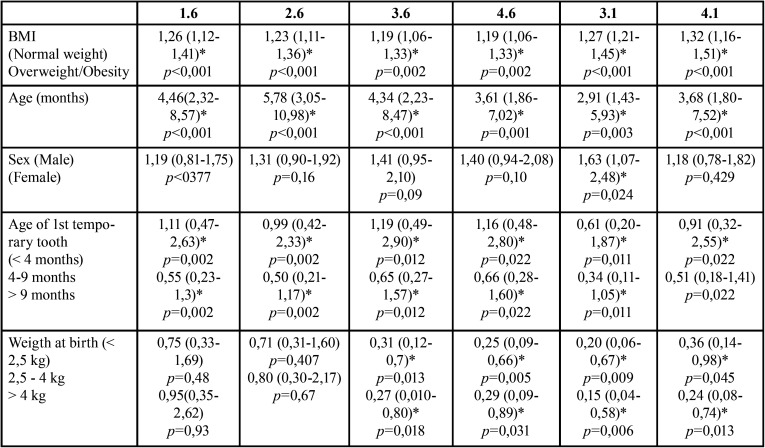
Multivariate model for BMI and dental eruption for each tooth including Odds ratio (OR) and 95% confidence Interval. Significant values are expressed as *.

Results herein indicate that low weight at birth is significantly associated to early dental eruption of 3.6, 4.6, 3.1 and 4.1. In other words, high BMI at birth reduces the probability of having the above-mentioned teeth at the same age.

There is also an association between the age of eruption of the first temporal tooth and the presence of the four molars and the two lower central incisors studied. The estimated ORs decrease as the appearance of the first temporary tooth is dilated. A delay on the first temporary tooth eruption retards the eruption of the permanent.

Breastfeeding and obesity family history don´t affect this process. The presence of obese siblings increases the possibility of an early dental eruption, to term pregnancy or prematurity neither influence dental eruption (Table 4).

**Table 4:**
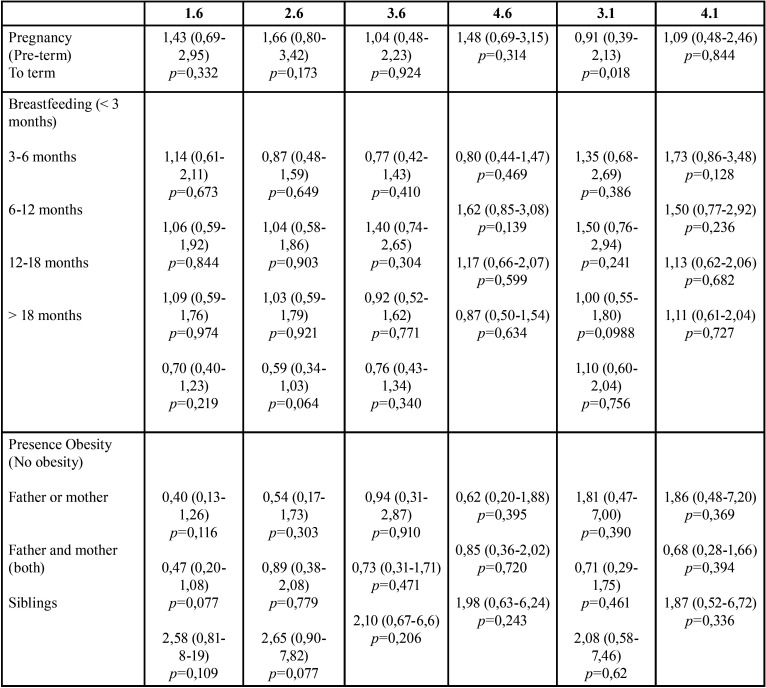
Multivariate model for BMI and dental eruption for each tooth including Odds ratio (OR) and 95% confidence Interval. Non-significant results.

It is noteworthy that even considering the age of first temporary tooth eruption, weight at birth and all the studied variables together; BMI and age are the master variables on early permanent dental eruption.

## Discussion

Childhood obesity and overweight is one of the most relevant challenges for science and society today. The number of cases with high BMI and the increasing trend is accompanied of many other systemic alterations associated to fat accumulation at early stages of child development (17). Overweight and obesity present different prevalence values according with age. Wong *et al*. report obese/overweight prevalence of 30,1% (5) and the prevalence found herein is a little different (23%). In a longitudinal study on 7 years old kids the probability to become overweight is 29,6% but at the age of 11 this probability increased to 45,5% (18).

One of the preliminary considerations is that dealing with methodology on the different studies. Only three worksdealing with BMI and early dental eruption are longitudinal studies (18-20). Longitudinal studies cover longer periods of time eg. four years providing more reliable data. These three longitudinal studies confirm a positive relation BMI and early dental eruption. Other factor is the clinical observation method to observe dental eruption (2,8,9,18,20-25). Similar reports have been performed with panoramic XRay according to Demirjian method (13,14). However only two studies used both clinical examination and radiological analysis (19)(26). Other studies obtained periapical XR images and genetic determinations from salivary samples (19,26).

Fitting with others, results herein indicate a positive relation within overweight and early teeth eruption (first molar and lower central incisor) (2,8,9,13,18-20,22). Hilgers *et al*. agree with our results indicating this positive correlation (13). Wong *et al*. reported that obese children had 1,9 more teeth compared to slim weight and 1,0 more than normal weight children (22). Similar results indicated that obese children presented 1,44 more teeth than normal weight children (9). Moreover, one longitudinal study indicated that 11 years old overweight children present 5 teeth more than slim weight (18). Longitudinal studies are more suiTable for this type of analysis since it allows to follow up the same subjects along the time.

Sinderova *et al*. found statistically significant results supporting the relationship Obesity and early dental eruption concretely for teeth 1.6, 4.1 and 3.6 (boys) and 4.1, 4.6 y 2.6 (girls) (1). Contrarily, Vijayakumar *et al*. reported a negative correlation, indicating a dental eruption delay on obese/overweight compared to normal weight kids (25). In contrast, Khan *et al*. did not conclude any negative or positive correlation between BMI and dental eruption (24).

We report herein the relationship between BMI and permanent tooth eruption however most of the works are focused on deciduous teeth. Alnemer *et al*. reported a positive correlation BMI and early deciduous teeth eruption without affecting permanent eruption (27).

In spite of BMI is positively related to permanent dental eruption, when analyzed at the same timepoint, we also report a significant correlation involving weight at birth and early permanent teeth eruption (for lower permanent molars and central incisors). Low weight at birth increases the probability to find permanent teeth at the age of 6. Fitting with this, Garmash *et al*., observed how high weight at birth is related to low average teeth eruption(28). In contrast, Kamakar *et al*. indicated the opposite, suggesting higher teeth eruption (29).

Tooth eruption is under both genetic and phenetic control. Several genes are closely related to dental eruption and some of them are also relevant in obesity (30). Among them, insulin-like grow factor (IGF2) results of interest since fatty cell overproduction regulate hormones and metabolic pathways finally increasing IGF2 and mineral metabolism plausibly affecting dental eruption (1,5). Congenic lack of leptin and its receptor has been demonstrated on early obese kids as cause of fat tissue development (31). Fibroblast growth factors play a relevant role in tooth development. These factors are also involved in metabolic energy processes as glucose or lipid metabolism (26).

Different authors indicate a close relationship between child obesity and early dental eruption. It seems plausible that different metabolic aspects are involved on this alteration. According to the progressive increase of childhood obesity/overweight worldwide and the amount of overweight-associated alterations, dental alterations must be more particularly observed on this group of people as part of the standard clinical practice.
